# Berberine Targets AP-2/hTERT, NF-κB/COX-2, HIF-1α/VEGF and Cytochrome-c/Caspase Signaling to Suppress Human Cancer Cell Growth

**DOI:** 10.1371/journal.pone.0069240

**Published:** 2013-07-15

**Authors:** Lingyi Fu, Wangbing Chen, Wei Guo, Jingshu Wang, Yun Tian, Dingbo Shi, Xiaohong Zhang, Huijuan Qiu, Xiangsheng Xiao, Tiebang Kang, Wenlin Huang, Shusen Wang, Wuguo Deng

**Affiliations:** 1 State Key Laboratory of Oncology in South China, Sun Yat-sen University Cancer Center, Guangzhou, China; 2 Institute of Cancer Stem Cell, Dalian medical University Cancer Center, Dalian, China; 3 State Key Laboratory of Targeted Drug for Tumors of Guangdong Province, Guangzhou Double Bioproduct Inc., Guangzhou, China; University of Pittsburgh Cancer Institute, United States of America

## Abstract

Berberine (BBR), an isoquinoline derivative alkaloid isolated from Chinese herbs, has a long history of uses for the treatment of multiple diseases, including cancers. However, the precise mechanisms of actions of BBR in human lung cancer cells remain unclear. In this study, we investigated the molecular mechanisms by which BBR inhibits cell growth in human non-small-cell lung cancer (NSCLC) cells. Treatment with BBR promoted cell morphology change, inhibited cell migration, proliferation and colony formation, and induced cell apoptosis. Further molecular mechanism study showed that BBR simultaneously targeted multiple cell signaling pathways to inhibit NSCLC cell growth. Treatment with BBR inhibited AP-2α and AP-2β expression and abrogated their binding on hTERT promoters, thereby inhibiting hTERT expression. Knockdown of AP-2α and AP-2β by siRNA considerably augmented the BBR-mediated inhibition of cell growth. BBR also suppressed the nuclear translocation of p50/p65 NF-κB proteins and their binding to COX-2 promoter, causing inhibition of COX-2. BBR also downregulated HIF-1α and VEGF expression and inhibited Akt and ERK phosphorylation. Knockdown of HIF-1α by siRNA considerably augmented the BBR-mediated inhibition of cell growth. Moreover, BBR treatment triggered cytochrome-c release from mitochondrial inter-membrane space into cytosol, promoted cleavage of caspase and PARP, and affected expression of BAX and Bcl-2, thereby activating apoptotic pathway. Taken together, these results demonstrated that BBR inhibited NSCLC cell growth by simultaneously targeting AP-2/hTERT, NF-κB/COX-2, HIF-1α/VEGF, PI3K/AKT, Raf/MEK/ERK and cytochrome-c/caspase signaling pathways. Our findings provide new insights into understanding the anticancer mechanisms of BBR in human lung cancer therapy.

## Introduction

Lung cancer ranks first among cancer related deaths in the world [1]. The incidence of non-small-cell lung cancer (NSCLC), a major form of lung cancer, has been increasing with significant mortality and morbidity. Treatment such as chemotherapy and radiation are the main therapy strategies of lung cancer [2]. In recent years, therapies selectively target cell signaling pathways, such as EGFR, VEGF, KRAS, BRAF, ALK, HER2, MET, TITF-1, p53, LKB1 and many others, not only provided a better understanding of NSCLC carcinogenesis, but also used as prognostic factors or targets for individualizing therapy [3]. However, the survival remains poor. Progress in lung cancer biology and genetics led to the development of small-molecule phytochemicals, especially phytochemicals extracted from Chinese herbs which have effects on cancer cell proliferation, angiogenesis and apoptosis [4]. Thus, optimization of use of conventional and novel therapeutic phytochemicals may improve the outcome of treatment for lung cancer.

Chinese herbs have been used widely and successfully for centuries in treating different kinds of diseases [5]. Phytochemicals from Chinese herbs have shown promise for the prevention of cancer with safety and efficacy [6]. Berberine (BBR) is an isoquinoline derivative alkaloid isolated from the rhizome, roots and stem bark of a number of Chinese herbs, the *Berberis* species, such as *Hydrastis* *canadensis* (goldenseal), *Cortex phellodendri* (Huangbai) and *Rhizomacoptidis* (Huanglian) [7]. Berberine has a long history of being used as a therapeutic agent to treat a variety of diseases, including cancer. It has been reported that BBR by exhibiting multiple pharmacological activities, including anti-inflammatory [8], anti-hypertensive [9], cholesterol-lowering [10], anti-diarrheal [11,12], anti-microbial [13,14] activities, and the anti-tumor effect of BBR was more and more emphasized in the past several decades [15,16]. BBR has been shown to exhibit anti-proliferation effects against cancer cells of different origins, including glioblastoma [17], melanoma [18], colon cancer [19], breast cancer [20,21], prostate cancer [22] and so on. In human lung cancer, it has been shown that BBR enhanced the cyto-toxicity of radiation in both *in vivo* and *in vitro* models via the induction of autophagy [23], and BBR exhibited a protective effect on radiation-induced lung injury through the intercellular adhesion molecular-1 and transforming growth factor-beta-1 [24]. BBR also effectively inhibited the motility and invasion ability of lung cancer cell line A549 in a dose- and time-dependent manner under non-cytotoxic concentrations via decreased productions of urokinase-plasminogen activator and matrix metalloproteinase-2 [25]. Furthermore, BBR was reported to inhibit growth and induce apoptosis in human lung cancer cells, and the administration of BBR by oral gavage inhibited the growth of tumor xenografts in athymic nude mice [26]. Although evidence of antitumor effects of BBR is expanding, uncertainty of the mechanisms of BBR in NSCLC still remains.

Human telomerase reverse transcriptase (hTERT) has shown to be an important component of human telomerase, which synthesizes telomeric DNA, lengthens chromosome ends and maintains chromosomal stability, finally leads to cellular immortalization [27]. hTERT is not expressed in most human somatic cells, but it is commonly overexpressed in a wide range of human cancers, including lung cancer [28]. The elevated expression of hTERT is necessary to transform normal human cells into cancer cells. Transcriptional regulation of hTERT gene is the major mechanism for cancer-specific activation of telomerase, and a number of factors have been identified to directly or indirectly regulate the hTERT promoter [29]. The activating enhancer-binding protein-2 (AP-2) has shown to transcriptionally control the expression of hTERT. The AP-2 transcription factor family contains a set of developmentally regulated, retinoic acid inducible genes composed of four related factors-AP2α, AP2β, AP2γ, and AP2δ [30]. By binding to the hTERT promoter, AP-2 factors exert their biological effects through activation of a number of tumor-related genes and signaling pathways, including hTERT, PI3K/Akt, and Raf/MEK/ERK [31].

Vascular endothelial growth factor (VEGF) has been recognized as one of the principal initiators in the development and progression of the vascularization system [32]. Pigment epithelium-derived factor (PEDF), a potent inhibitor of angiogenesis as well as a neuro-protective factor, counterbalances the effect of VEGF [33]. VEGF and PEDF both possess multiple biological activities and functions and they have an inverse relationship with each other especially in cancer [34]. The expression of VEGF and PEDF is regulated by a body of external factors, of which hypoxia is the best characterized mediator. Hypoxia-inducible factor-1α (HIF-1α) is a transcription factor which plays a crucial role in carcinogenesis. The activity of HIF-1α is upregulated by a variety of non-hypoxic signals, including the activation by several oncogenic pathways such as Src, HER-2, Ha-Ras, and mitogen-activated protein kinase (MAPK) signaling [35]. By binding to the hypoxia-responsive elements on VEGF promoter, HIF-1 leads to the transcriptional activation of the VEGF gene [36].

COX-2 is an inducible enzyme that converts arachidonic acid to prostaglandins, and it is commonly overexpressed in a wide range of human cancers [37]. The increased expression of COX-2 protein and the sequencial PGE2 production have been shown to significantly enhance carcinogenesis and inflammatory reactions by upregulating EGFR, PI3K, and ERK1/2 signaling [38]. The COX-2 expression is transcriptionally controlled by the binding of multiple transactivators and coactivators to the corresponding sites located in its promoter. Among the known several regulatory elements distributing in the core promoter region of COX-2 transcription start site, NF-κB binding site is essential for COX-2 promoter activity [39,40].

The PI3K/AKT and Raf/MEK/ERK are two classical cell signaling pathways and plays a key role in the regulation of cell gene expression, growth survival. Abnormal PI3K/AKT and Raf/MEK/ERK signaling may lead to increased or uncontrolled cell proliferation, resistance to apoptosis and to chemotherapy, radiotherapy, and targeting therapies in tumors. The activation of HIF-1 protein could be facilitated by PI3K/AKT and Raf/MEK/ERK signaling. As the upstream signaling molecules of HIF-1 protein, ERK might also play its role in mediating the effect of BBR on the inhibition of cells growth [41].

The induction of apoptosis is considered as one of the possible mechanisms of inhibition of cancer development. Caspase activation plays a central role in the execution of apoptosis. The activated caspases cleave a variety of target proteins, thereby disabling important cellular processes and breaking down structural components of the cell. The release of cytochrome-c from the mitochondrial inter-membrane space into the cytosol is the precondition of caspase-dependent apoptosis pathway. Cytochrome c binds to Apaf-1 in the absence of dATP, and the complex binds pro-caspase-9 to form apoptosome, which cleaves the pro-caspase to caspase 9, and in turn activates the effector caspase-3 [42].

In this study, we investigated the detailed underlying mechanisms of BBR in inhibiting NSCLC cell growth in NSCLC *in vitro*. Our findings provide new insights into exploring the potential therapeutic strategies and novel targets for human lung cancer.

## Results

### BBR changed cell morphology and inhibited cell migration

**Figure 1:**
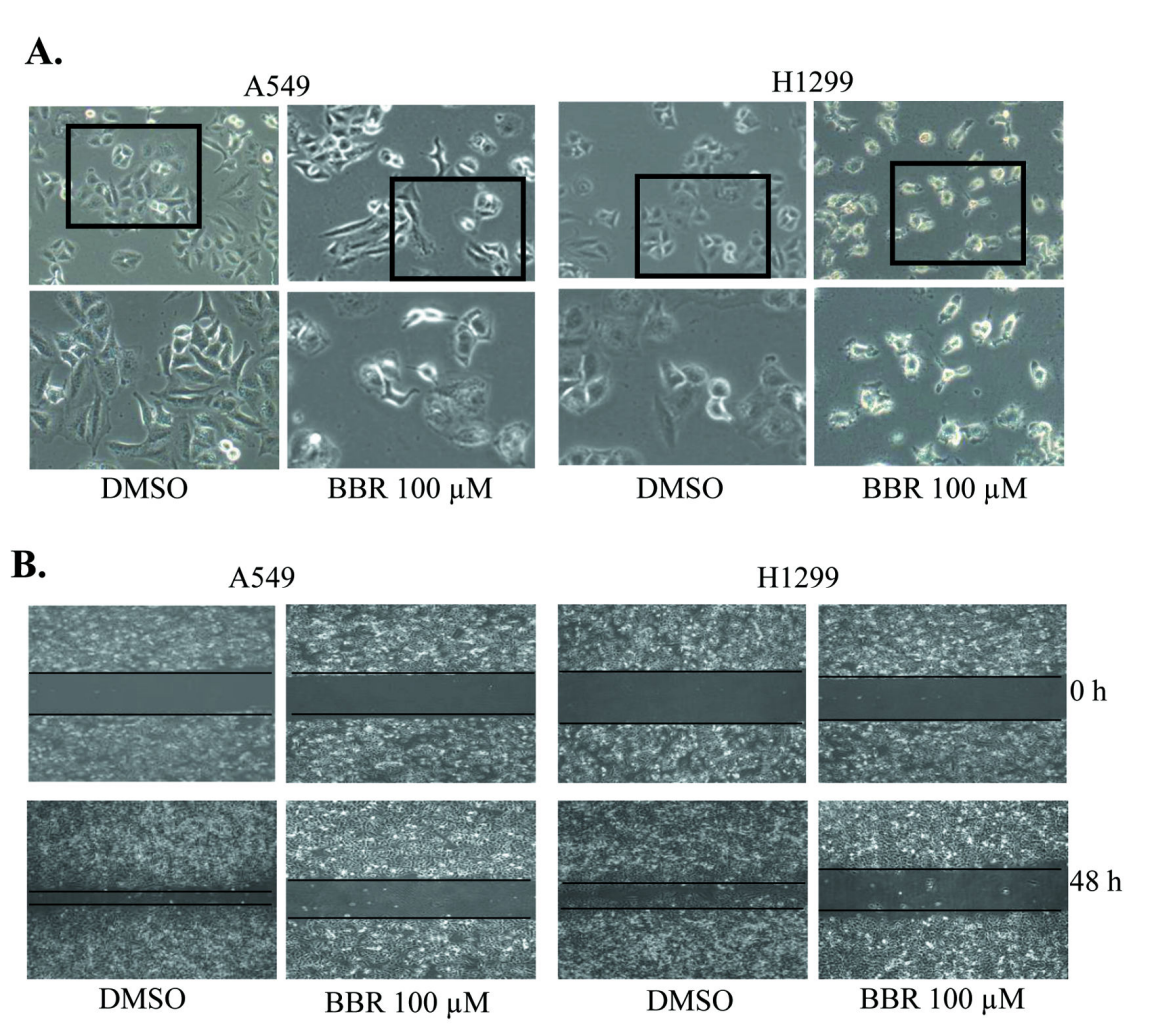
BBR changed cell morphology and inhibited migration. (**A**) The changes in cell morphology and spreading in A549 and H1299 cells treated with 100 µM BBR for 48 h were observed and cells were photographed using a microscope fitted with digital camera. (**B**) Cell migration was analyzed by a wound-healing assay. Cells were grown to full confluency. The cell monolayers were wounded with a sterile pipette tip, and washed with medium to remove detached cells from the plates. Cells were left either untreated or treated with 20 µM BBR. After 48 h, the wound gap was observed and cells were photographed.

We first analyzed the effect of BBR on cell morphology in A549 and H1299 cells. Treatment with BBR at 100 µM BBR effectively reduced cell-to-cell contact and led to a lower spreading with fewer formation of ﬁlopodia by comparison with the DMSO control groups (Figure 1A). We also tested the effect of BBR on cell migration by employing a wound-healing assay. As shown in Figure 1B, the part of the wounding space between cell layers after making a scratch was occupied completely by the migrating cells after 48 h in the control group. However, the empty space of the cells was not occupied by the migrating cells treated with 20 µM BBR. These results demonstrated the potentials of BBR in changing cell morphology and inhibiting cell migration in lung cancer cells

### BBR suppressed cell proliferation and colony formation

**Figure 2:**
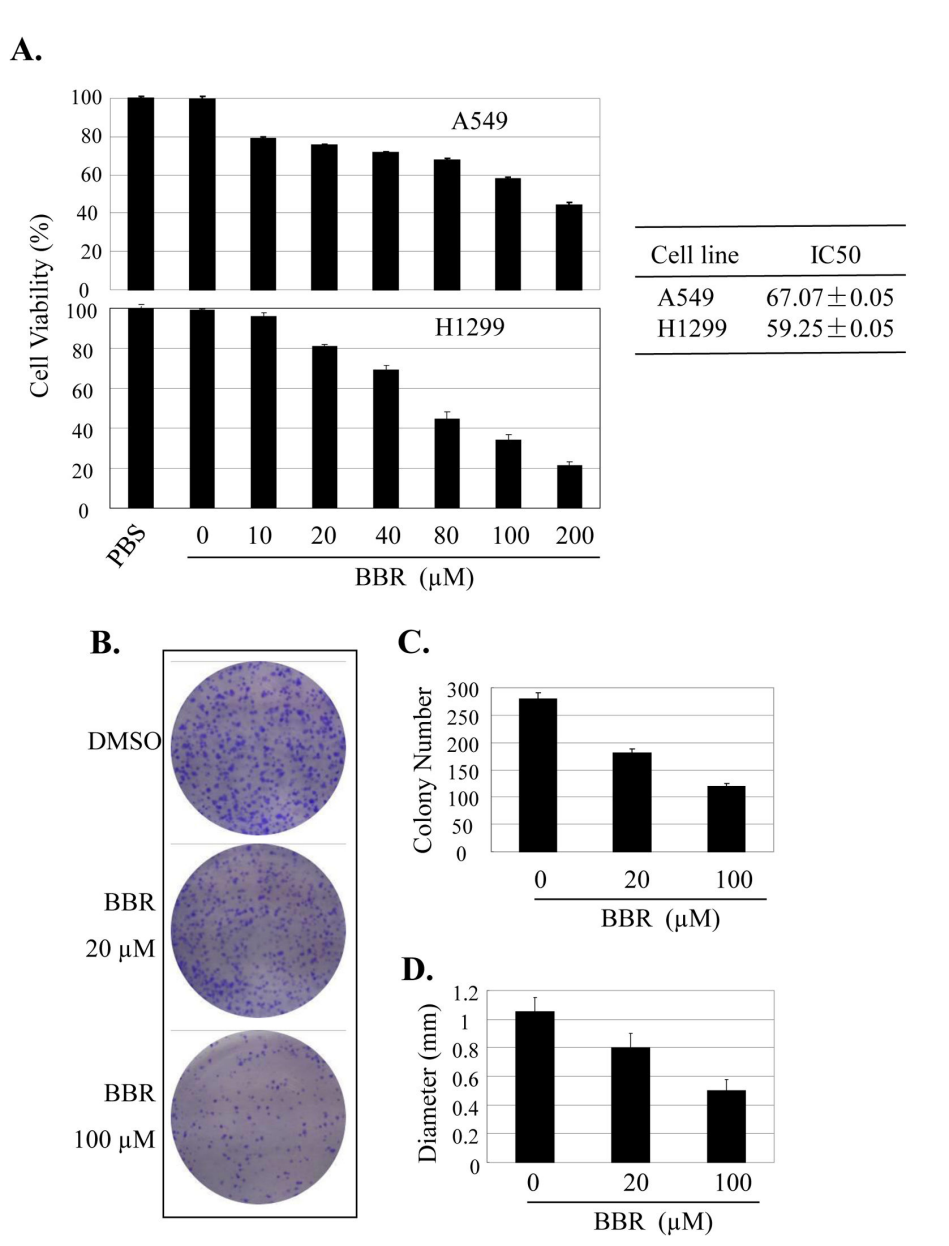
BBR suppressed lung cancer cell proliferation and colony formation. (**A**–**C**) Human A549 and H1299 cells were treated with BBR at the indicated doses. At 48 hours after treatment, the cell viability was determined by a MTT assay. The IC50 values of BBR for A549 and H1299 cells were calculated (**A**). The tumor cell A549-induced colony formation was also analyzed (**B**), and the colony formation rate (**C**) and colony size (**D**) were calculated. Cells treated with DMSO were used as the referent group with cell viability set at 100%. The percent cell viability in each treatment group was calculated relative to cells treated with DMSO vehicle control. The data are presented as mean ± SD of three tests. *, P < 0.05, significant differences between treatment groups and DMSO control groups.

Berberine has been shown to induce growth inhibition and apoptosis of non-small cell human lung cancer cells via p53 signaling pathway [43]. We next quantitatively analyzed the effect of BBR on cell proliferation in lung cancer A549 and H1299 cells by MTT assay. Treatment with BBR at the dose of 10 µM to 200 µM inhibited cell viability in a dose-dependent manner. The IC_50_ values of BBR for A549 and H1299 cells are 67.1 µM and 59.2 µM, respectively (Figure 2A). We also tested the effects of BBR on tumor cell clonogenicity in A549 cells (Figure 2B). Treatment with BBR markedly inhibited colony formation, resulting in a significant decrease at both colony formation ratio (Figure 2C) and colony size (Figure 2D).

### BBR inhibited AP-2/hTERT signaling

**Figure 3:**
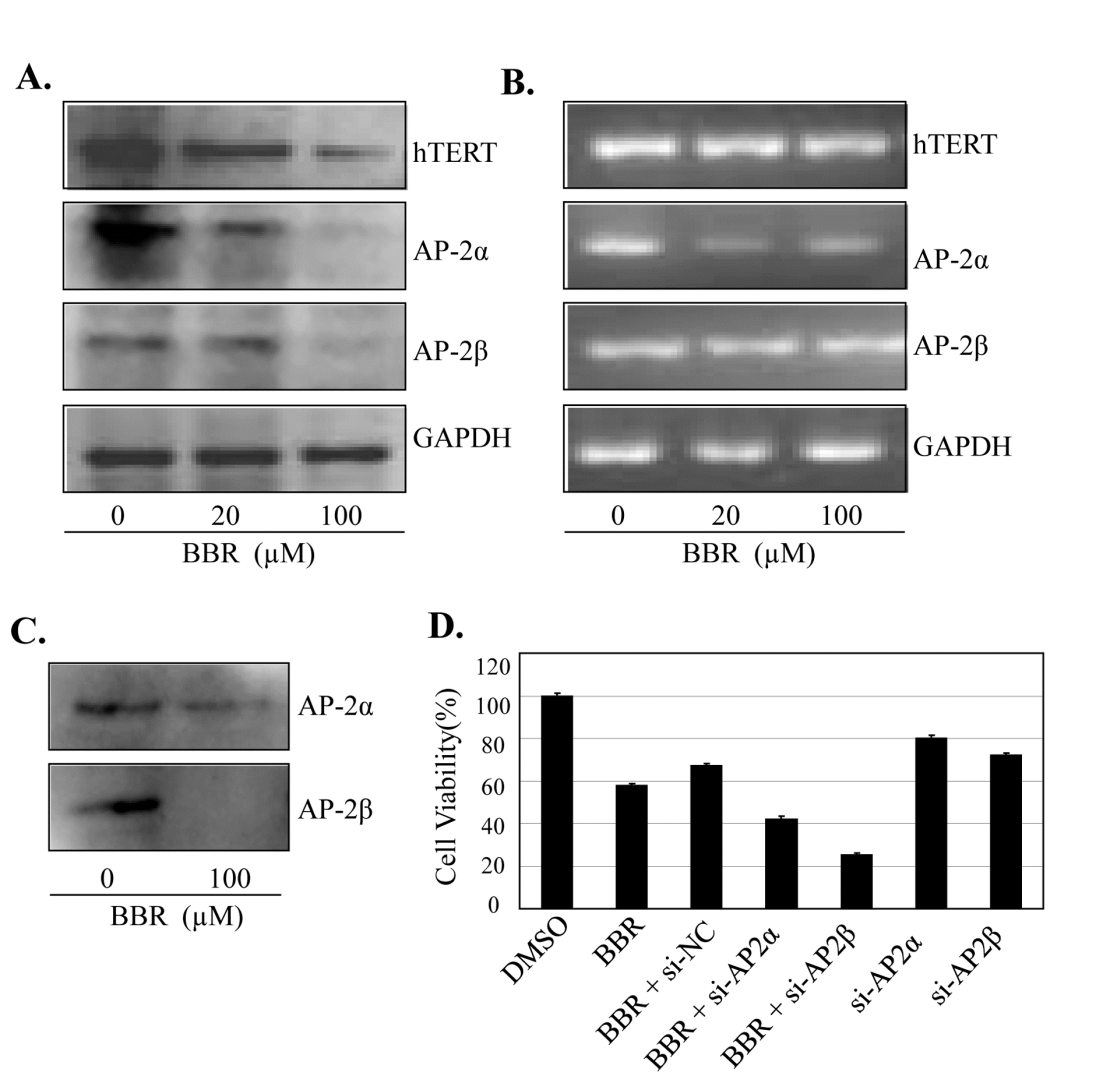
BBR inhibited AP-2/hTERT signaling. (**A**–**C**) Human NSCLC A549 cells were treated with BBR at the indicated doses. At 48 hours after treatment, the AP-2 and hTERT proteins (**A**) and mRNA (**B**) were analyzed by Western blotting and RT-PCR, respectively. GAPDH were used as controls for sample loading. The binding of AP-2 to hTERT promoter probe (**C**) was analyzed by a streptavidin-agarose pulldown assay. (**D**) A549 cells were transfected with an AP-2 siRNA or an AP-2-expressing vector for 24 hours, and then treated with BBR (100 µM). At 48 hours after treatment, protein expression and cell viability were determined by Western blot and MTT assay, respectively. The percent cell viability in each treatment group was calculated relative to cells treated with the vehicle control. The data are presented as the mean ± SD of three separate experiments. *, P < 0.05, significant differences between treatment groups and DMSO control groups.

hTERT has been regarded as the hallmark of carcinogenesis and the expression of hTERT is tightly controlled by transcriptional factor AP-2. To determine whether BBR targets the AP-2/hTERT signaling, we treated A549 cells with BBR (20 µM or 100 µM) and examined the expression of AP-2α, AP-2β and hTERT proteins and mRNAs by Western blot and RT-PCR, respectively. Treatment with BBR led to a marked inhibition of the expression of AP-2α, AP-2β and hTERT at protein (Figure 3A) and mRNA levels (Figure 3B).

Since hTERT expression is tightly controlled by the binding activity of AP-2 family on hTERT promoter, we next performed streptavidin-agarose pull-down assay to examine the effect of BBR on AP-2α and AP-2β binding activities at hTERT promoter. As shown in Figure 3C, BBR treatment effectively abrogated AP-2α and AP-2β binding to the biotin-labeled hTERT promoter DNA probe (Figure 3C).

To confirm the role of AP-2 signaling in BBR-mediated cell proliferation inhibition, we transfected A549 cells with AP-2α or AP-2β siRNA (100 nM) and then treated them with BBR (100 µM) for 48 h. The results showed that transfection with AP-2α or AP-2β siRNA effectively down-regulated the expression of AP-2α or AP-2β protein and considerably inhibited cell viability (Figure 3D). Knockdown of AP-2α or AP-2β by siRNA also markedly augmented the BBR-mediated inhibition of cell viability compared with the transfection with the control siRNA (si-NC) (Figure 3D). These results confirm that the effect of BBR on cell proliferation inhibition is mediated at least in part through AP-2/hTERT signaling pathway in lung cancer cells.

### BBR inhibited NF-κB/COX-2 signaling

**Figure 4:**
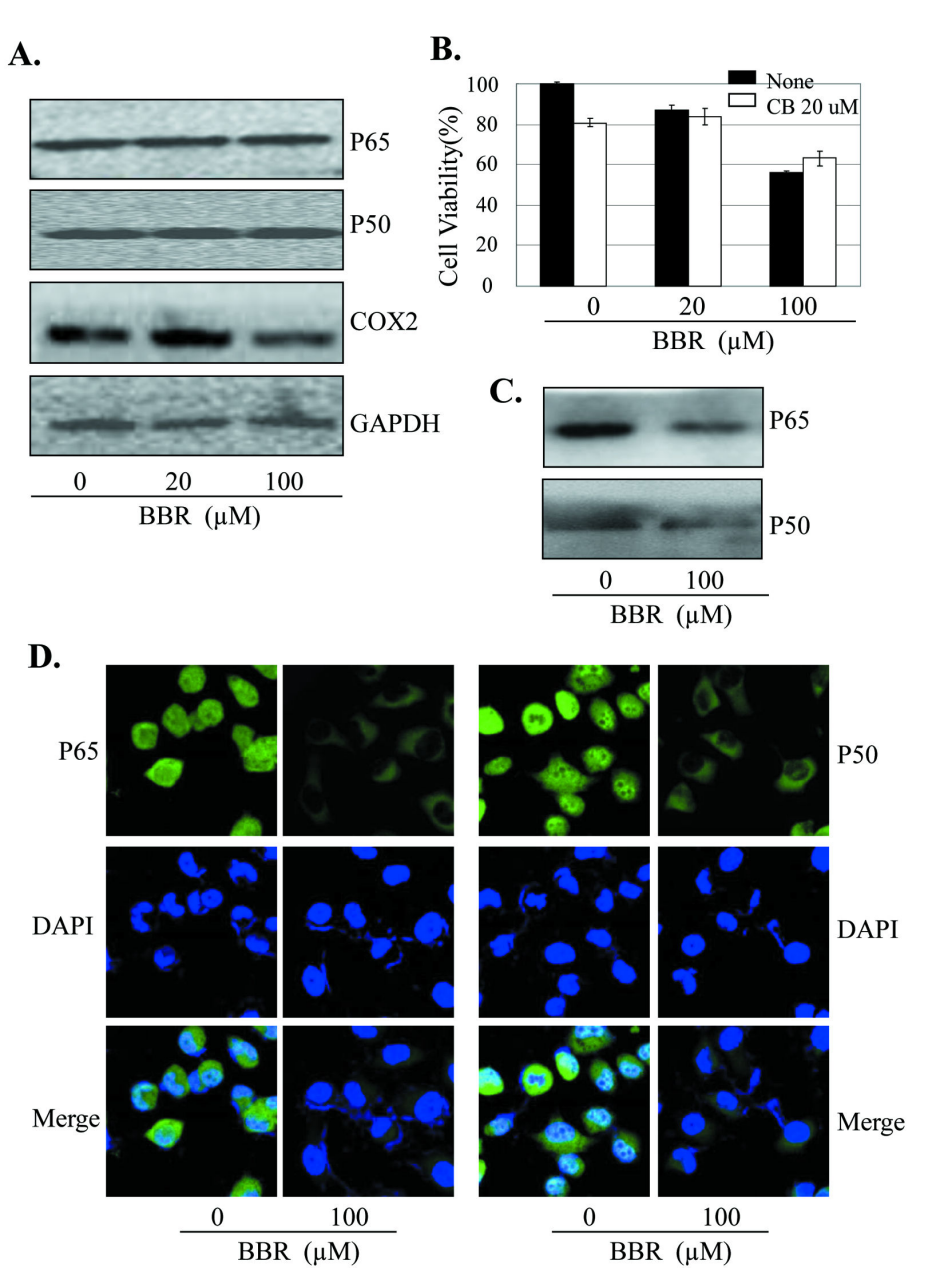
BBR inhibited NF-κB/COX-2 signaling. (**A**) Human A549 cells were treated with BBR at the indicated doses. At 48 hours after treatment, the COX-2 protein was analyzed by Western blotting. GAPDH were used as controls for sample loading. (**B**) A549 cells were pretreated with the COX-2 selective inhibitor celecoxib (CB, 20 µM) for 24 hours, and then treated with BBR (20 µM). At 48 hours after treatment, cell viability was determined by MTT analysis. The percent cell viability in each treatment group was calculated relative to cells treated with the vehicle control. (**C**) A549 cells were treated with BBR at the indicated doses. At 48 hours after treatment, the binding of p50 and p65 to COX-2 promoter probe was analyzed by a streptavidin-agarose pulldown assay. (**D**) A549 cells were treated with BBR (100 nM). At 48 hours after treatment, the effect of BBR on NF-κB p65 and p50 translocation was analyzed by immunofluorescence assay. The data are presented as the mean ± SD of three separate experiments. *, P < 0.05, significant differences between treatment groups and DMSO control groups.

High expression of COX-2 is implicated in cancer cell growth, migration and angiogenesis. To determine the effects of BBR on COX-2 signaling in lung cancer cells, we next analyzed the expression of COX-2 protein in A549 cells treated with BBR by Western blot. Treatment with BBR at the dose of 20 µM did not significantly inhibited COX-2 protein expression, while the dose of 100 µM markedly decreased the COX-2 protein expression in A549 cells (Figure 4A).

The expression of COX-2 is tightly regulated by the binding activity of p50/p65 NF-κB on COX-2 promoter structure. We next determined whether the BBR-induced inhibition of tumor cell proliferation and COX-2 expression is mediated by inhibition of the binding of NF-κB to COX-2 promoter in A549 cells. Streptavidin-agarose pull-down assay showed that BBR at the dose of 100 µM markedly inhibited NF-κB p50 and p65 binding to COX-2 promoter probe as compared with the control group (Figure 4B). By contrast, BBR at the dose of 20 and 100 µM did not affect p50 and p65 protein levels in whole cell lysates (Figure 4A).

The translocation of NF-κB p65 and p50 in cell nuclei and cytoplasma plays a key role in regulate COX-2 gene expression. We next performed immunofluorescence assay to evaluate the effect of BBR on NF-κB p65 and p50 translocation in A549 cells. Constitutive translocation of NF-κB p65 and p50 to the cell nuclei was detected (Figure 4C). Treatment with BBR (100 µM) effectively promoted translocation of the NF-κB p65 and p50 from cell nuclei to cytoplasma. The results suggest that the inhibition of tumor cell growth by BBR might be also mediated by modulating the NF-κB/COX-2 signaling pathway in lung cancer cells.

### BBR inhibited HIF-1α/VEGF, PI3K/AKT, Raf/MEK/ERK signaling

**Figure 5:**
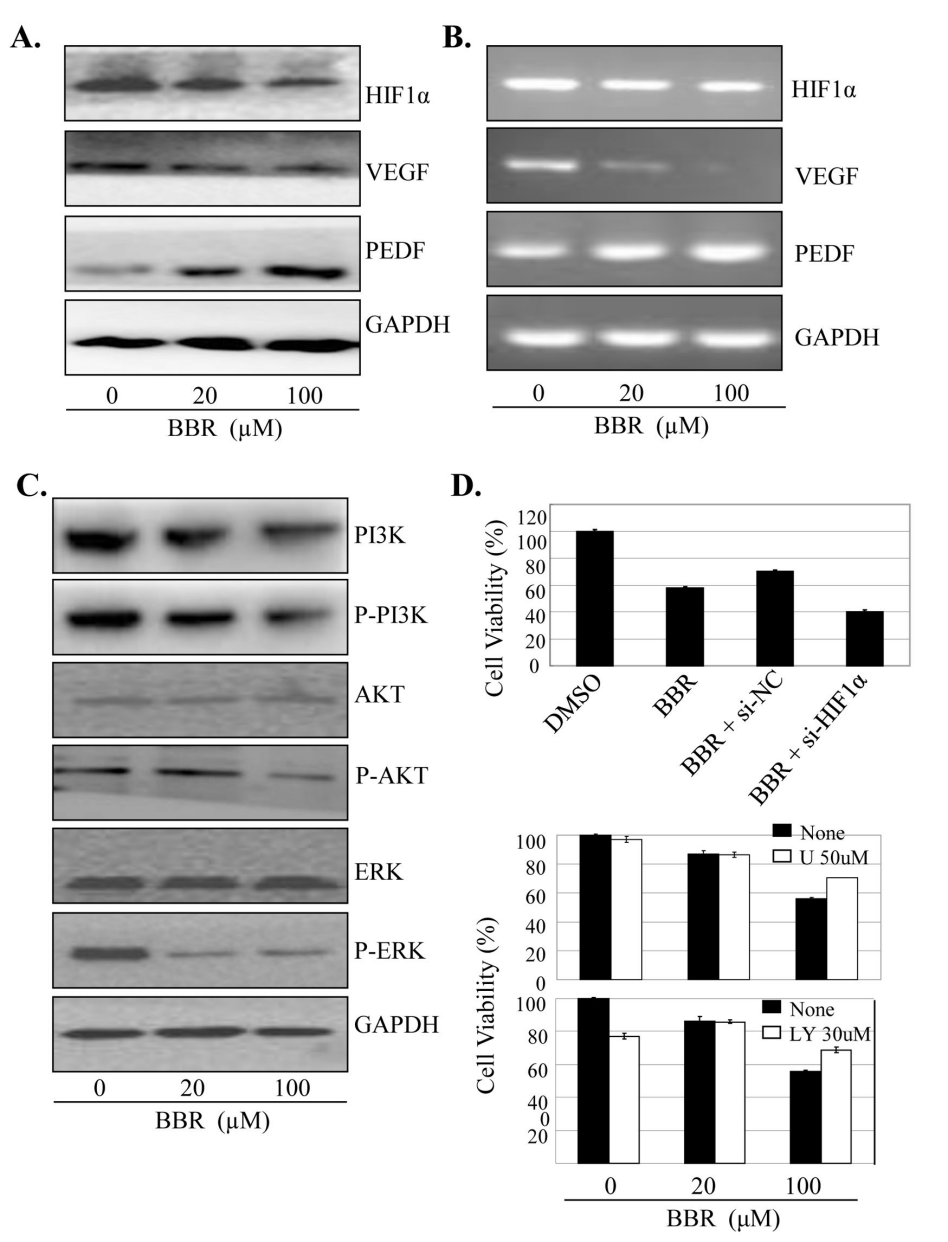
BBR inhibited BBR inhibited HIF-1α/VEGF Raf/MEK/ERK and PI3K/AKT signaling. A549 cells were treated with BBR at the indicated doses. At 48 hours after treatment, the expression of HIF-1α, VEGF and PEDF at protein (**A**) or mRNA levels (**B**), as well as the total and phosphorylated Akt and ERK1/2 proteins (**D**), were determined. A549 cells were treated with the HIF-1α-specific siRNA (si-HIF) (**C**) or the Akt-selective inhibitor LY294002 (LY,30 µM) or the ERK-selective inhibitor U0126 (U, 50 µM) (E) for 24 hours, respectively, and then treated with BBR. At 48 hours after treatment, cell viability was determined by MTT analysis. The percent cell viability in each treatment group was calculated relative to cells treated with the vehicle control. The data are presented as the mean ± SD of three separate experiments. *, P < 0.05, significant differences between treatment groups and DMSO control groups.

The HIF-1α/VEGF/PEDF signaling is closely associated with cancer cell growth, migration and angiogenesis. We next determined the effect of BBR on the expression of HIF-1α, VEGF and PEDF at protein and mRNA levels in lung cancer cells by RT-PCR and Western blot, respectively. Treatment of A549 cells with BBR significantly inhibited the expression of HIF-1α and VEGF, but not PEDF, at protein (Figure 5A) and mRNA levels (Figure 5B).

To validate the effects of BBR on HIF-1α/VEGF/PEDF signaling, we transfected A549 cells with HIF-1α siRNA (100 nM) and then treated them with BBR (100 µM) for 48 h. As shown in Figure 5C, knockdown of HIF-1α by siRNA considerably increased the BBR-mediated inhibition of cell proliferation as compared with the transfection with the control siRNA. These results confirm that the effect of BBR on cell proliferation inhibition is also mediated partially through HIF-1α/VEGF/PEDF signaling pathway in lung cancer cells.

The PI3K/AKT and Raf/MEK/ERK signaling plays an important role in regulating tumor cell proliferation and survival. To determine whether the BBR-mediated cell growth inhibition is also through the inactivation of the PI3K/AKT and Raf/MEK/ERK signaling pathway, we analyzed the effect of BBR on the phosphorylation of Akt and ERK in A549 cells by Western blot. As shown in Figure 5D, treatment with BBR significantly decreased the levels of the phosphorylated Akt and ERK1/2 proteins, whereas the levels of total Akt and ERK1/2 protein did not change.

### BBR activated caspase-dependent apoptotic pathway

**Figure 6:**
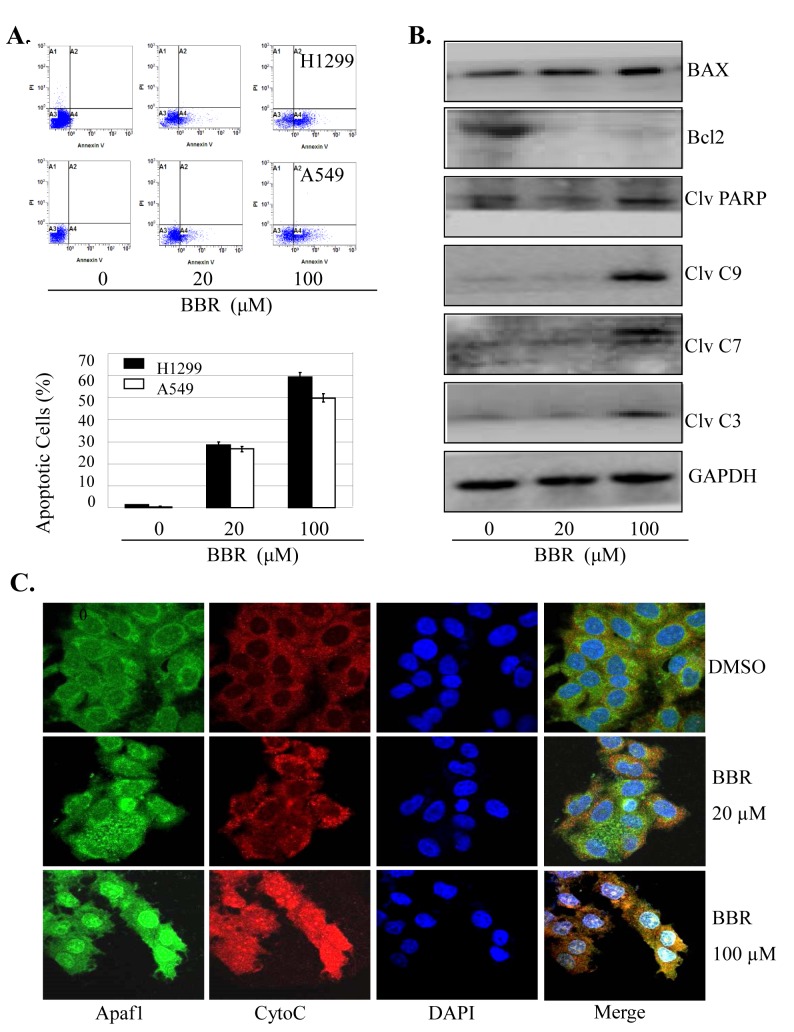
BBR activated caspase-dependet apoptotic pathway. A549 and H1299 cells were treated with BBR (20 µM or 100 µM). At 48 hours after treatment, the apoptosis was determined by a FACS analysis (**A**). The levels of the cleaved caspase-3, 7, 9, cleaved PARP, BAX and Bcl-2 proteins in A549 cells were analyzed by Western blot (**B**). The release of Cyt-c in A549 cells was analyzed by immunofluorescence imaging analysis to monitor Cyt-c release from the inter-mitochondrial space into the cytosol (**C**). The apoptosis are represented by relative percentages of apoptotic cells versus that in DMSO-treated cells. *, P<0.05, significant differences between the BBR-treated groups and the DMSO-treated groups.

We also determined whether the enhancement of cell growth inhibition induced by BBR is associated with the increase of apoptosis in lung cancer cells. Treatment with BBR at the doses of 20 µM and 100 µM induced 26% and 50% apoptotic cells in A549 and 28% and 60% apoptotic cells in H1299 cells (Figure 6A). To confirm the effect of BBR on apoptosis, we next detected the expression of some pro-apoptotic and anti-apoptotic proteins, caspase-3/7/9, PARP, BAX and Bcl-2 in A549 cells by Western blot analysis. BBR markedly increased the expression levels of cleaved caspase-3/7/9, cleaved PARP and BAX proteins, but decreased the Bcl-2 protein levels, as compared with the control group (Figure 6B).

Cytochrome-c (Cyto-c) release is an important event in the caspase-dependent apoptosis pathway. We next performed immunofluorescence imaging (IF) analysis to monitor changes in the subcellular localization of Cyto-c in A549 cells to determine whether BBR could induce Cyto-c release. Treatment with BBR (20 µM or 100 µM) markedly triggered the release of Cyto-c from the inter-mitochondrial space into the cytosol (Figure 6C). These results demonstrate that BBR may facilitate the downstream Cyto-c-dependent apoptosome assembly and caspase activation in the cytosol in lung cancer cells.

## Discussion

In this study, we evaluated the response of human lung cancer cells A549 and H1299 to BBR, an isoquinoline derivative alkaloid isolated from Chinese herbs. We found that BBR enhanced cell growth inhibition and apoptosis induction in a dose-dependent manner, while these effects were not observed in normal human bronchial epithelial cells (data not shown). Our results showed that the antitumor effect of BBR is mediated through modulation of the AP-2/hTERT, HIF-1α/VEGF/PEDF, NF-κB/COX-2, PI3K/Akt, Raf/MEK/ERK, caspase/cytochrome C and BAX/Bcl-2-dependent signaling.

hTERT has been regarded as the landmark of carcinogenesis. The AP2/hTERT signaling was shown to be important in non-small cell lung cancer [44]. The inhibition of hTERT expression was found to contribute to prevent proliferation and angiogenesis and to induce apoptosis of cancer cells [45]. In our study, we demonstrated that the effect of BBR on tumor cell proliferation inhibition is mediated partially through the AP-2/hTERT signaling. Treatment with BBR suppressed the expression of AP-2a and AP-2b and reduced their protein abundance in cell nuclei, thereby decreasing the binding of AP-2a and AP-2b to hTERT promoter and down-regulating the expression of hTERT in the BBR-treated cells**.**

The increased ratio of VEGF/PEDF is required for angiogenesis and tumor growth [46]. The HIF-1α/VEGF/PEDF pathway exists as a critical step in lung cancer angiogenesis and tumor metastasis [47]. Our study showed that the BBR inhibited HIF-1α expression, thereby inhibited the VEGF/PEDF ratio in lung cancer cells. It is possible that BBR inhibited HIF-1α protein accumulation in lung cancer cells, and then suppressed the expression of the related genes which are involved in tumor cell proliferation. Thus, our results show that HIF-1α signaling contributes, at least in part, to BBR-induced cell growth inhibition.

COX2 plays a key role in early stages of lung carcinogenesis [48]. Here, we demonstrated that BBR played its anti-proliferative role partially through inhibiting COX-2 expression. We found that BBR inhibited COX-2 expression not only at protein level but also at mRNA level, suggesting that BBR could exert antitumor effect partially through suppression of COX-2 signaling. We also found that the inhibition of COX-2 expression by treatment of BBR is partially mediated by stimulating NF-κB translocation from nuclear to cytosol and by inhibiting the binding of NF-κB to COX-2 promoter.

The PI3K/AKT and Raf/MEK/ERK signaling play a key role in the regulation of cell gene expression, growth survival. The relationship of Raf/MEK/ERK and PI3K/AKT to lung cancer is still an intense research area nowadays [49,50]. Our research demonstrated the important role of PI3K/AKT and Raf/MEK/ERK signaling pathway in BBR-mediated cell proliferation inhibition. BBR might function through inactivating PI3K/AKT and Raf/MEK/ERK signaling pathway, inhibiting the phosphorylation of Akt and ERK proteins, and downregulating HIF-1α signaling to inhibit tumor cell growth.

Apoptosis play a crucial role in the response of cancer to chemotherapy and radiation therapy. In this study, we demonstrated that the induction of apoptosis in human lung cancer cells by BBR was mediated by cytochrome-c and caspase-dependent apoptosis pathways. We found that BBR induced the activation of caspase and PARP proteins, and promoted the release of cytochrome-c from mitochondria to cytosol. Our results therefore suggest the antitumor effect of BBR in lung cancer cells is associated with the increased activation of the cytochrome-c and caspase-dependent apoptotic pathway.

In summary, we demonstrated that BBR exhibited anti-proliferative and pro-apoptotic activities in NSCLC cells through simultaneous modulation of the AP-2/hTERT, HIF-1α/VEGF, NF-κB/COX-2, Raf/MEK/ERK, PI3K/AKT and caspase/cytochrome-c signaling pathways. These findings provide new insights in exploring the new potential therapeutic strategies and novel targets for lung cancer treatment.

## Materials and Methods

### Cell culture

The human lung cancer cell lines A549 and H1299 were obtained from American Type Culture Collection (ATCC, Manassas, VA). Cells were cultured as monolayers in RPMI 1640 culture media supplemented with 10% heat-inactivated fetal bovine serum, 100 µg/ml penicillin, and 100 µg/ml streptomycin and maintained in an incubator with a humidified atmosphere of 95% air and 5% CO_2_ at 37 °C.

### Reagents and antibodies

Berberine (BBR), U0126, LY294002 and celecoxib were purchased from Sigma (St. Louis, MO) and dissolved in a small amount of DMSO before addition to the complete cell culture medium. Streptavidin-agarose was purchased from Sigma (St. Louis, MO). Antibodies to GAPDH, VEGF, PEDF, HIF-1α, p50, p65, COX-2 were obtained from Santa Cruz Biotechnology (Santa Cruz, CA). Antibodies against AP-2α, AP-2β, hTERT, cytochrome-c, PARP, caspase-3/7/9, BAX, Bcl-2, Akt, ERK1/2, phosphrylated Akt or ERK1/2 were purchased from Cell Signaling (Beverly, MA).

### Wound-healing assay

The wound-healing assay was performed to detect cell migration. The cells were grown to full confluency in six-well plates and incubated overnight in starvation medium. Cell monolayers were wounded with a sterile 100 µL pipettetip, washed with starvation medium to remove detached cells from the plates. Cells were treated with indicated doses of BBR in full medium and kept in a CO_2_ incubator. After 48 h, medium was replaced with PBS, the wound gap was observed and cells were photographed using an Olympus microscope fitted with digital camera.

### Cell viability assay

Cell viability was determined using MTT assay (Roche Diagnosis, Indianapolis, IN). Briefly, lung cancer cell lines were seeded at 4×103 cells/well in 96-well plates. Cells were cultured for overnight, and then the cells were changed to fresh medium containing various concentrations of BBR dissolved in DMSO (final concentration, 0.1%). After the cells were incubated for 48 h, the growth of cells was measured. The effect on cell viability was assessed as the percent cell viability compared with vehicle-treated control cells, which were arbitrarily assigned 100% viability. The BBR concentration required to cause 50% cell growth inhibition (IC50) was determined by interpolation from dose–response curves.

### Anchorage independent colony formation assay

A549 cells plated in 6-well plates were treated with BBR. After 24h, cells were washed with PBS and trypsinized. Then cells (8×10^3^/ml) were mixed in 1.0 ml of 0.3% McCoy’s 5a agar containing 10% FBS. The cultures were maintained in a 37°C, 5% CO_2_ incubator for 14 days. The medium was discarded and the cells were carefully washed with PBS twice. After being fixed with 4% paraformaldehyde for 15min, the cells were stained with 0.1% crystal violet for 15 min before washing with tap water and air-drying. The colonies with more than 50 cells were counted with an ordinary optical microscope. The colony formation rate was calculated with the following formula: Plate colony formation inhibitory ratio = (number of colonies treated with BBR/number of cells inoculated) ×100%.

### Apoptosis assay

Apoptosis were measured by flow cytometry using Annexin-V staining-based fluorescence-activated cell sorter (FACS). In brief, cells plated in 6-well plates were treated with BBR. At 48 h after treatment, cells were collected and washed once with cold PBS, and subsequently stained with Annexin V (Invitrogen, Carlsbad, CA). Stained cells were analyzed by flow cytometry.

### Western blot analysis

Cell lysate proteins were separated by electrophoresis in a 10% sodium dodecyl sulfate-polyacrylamide minigel (SDS-PAGE) and electrophoretically transferred to a PVDF membrane. Western blots were probed with the specific antibodies. The protein bands were detected by enhanced chemiluminescence.

### Reverse transcription-polymerase chain reaction (RT-PCR)

Total cellular RNA was extracted with Tri-Zol reagent (Life Technologies, Glasgow, UK) according to the manufacturer’s instructions. Total RNA was reverse-transcribed by using the Superscript™-III kit (Invitrogen, Carlsbad, CA). PCR analysis was performed on aliquots of the cDNA preparations to detect gene expression. The amplified products were visualized on 1% agarose gels. PCR conditions were 4 min at 94°C followed by 35 cycles (25 for GAPDH): 30 seconds at 94°C, 30 seconds at 60°C, and 1 min at 72°C.

### Transfection

The transfections of siRNAs or expressing vectors were performed by Lipofectamine 2000 reagent according to the manufacturer’s protocol (Invitrogen, Carlsbad, CA).

### DNA-protein binding by streptavidin-agarose pulldown assay

Binding of AP-2α, AP-2β or p65, p50 NF-κB to hTERT core promoter or COX-2 core promoter probes were determined by a streptavidin-agarose pulldown assay. A biotin-labeled double-stranded probe corresponding to hTERT or COX-2 promoter sequence was synthesized. The binding assay was performed by mixing 400 µg of nuclear extract proteins, 4 µg of the biotinylated DNA probe and 40 µl of 4% streptavidin-conjugated agarose beads at room temperature for 1 h in a rotating shaker. Beads were pelleted by centrifugation to pull down the DNA-protein complex. After washing, proteins in the complex were analyzed by immunoblotting using antibodies (1 µg/ml each) specific for AP-2α, AP-2β or p65, p50 NF-κB. The mixture was incubated at room temperature for 1 h with shaking, and centrifuged to pull down the DNA-protein complex. DNA-bound AP-2α, AP-2β or p65, p50 NF-κB protein was dissociated and analyzed by Western blotting. A non-immune rabbit IgG (1 µg/ml) was also used as negative controls.

### Confocal immunofluorescence

For confocal microscopy analysis, cells grown on chamber slides were washed in PBS, and fixed and got partial permeabilisation with 4% para formaldehyde for 15 min at room temperature. The samples were pretreated with 10% bovine serum albumin (BSA) in PBS for 30 min. Antibodies against cytochrome-c, p65, p50 in the blocking solution were added to the sample and incubated for overnight at 4°C. Nonimmune IgG was included as controls. Following five 5-min washes with PBS, fluorescein isothiocyanate- and rhodamineconjugated secondary antibodies were added in blocking solutions and incubated for 1 hour. After five additional 5-min washes, samples were examined with a Zeiss LSM700 confocal microscope, and images were processed with Zen2010 software. More than 100 cells were inspected per experiment, and cells with typical morphology were presented.

### Statistical analysis

Analysis of variance and Student’s t test were used to compare the values of the test and control samples. P<0.05 was considered to a statistically significant difference. SPSS6.0 software was used for all statistical analysis. The significance was analyzed by the paired t test. All the experiments were done three times, and mean values and standard deviation were calculated.
